# Relationship Between Loss of Y Chromosome and Urologic Cancers: New Future Perspectives

**DOI:** 10.3390/cancers16223766

**Published:** 2024-11-08

**Authors:** Pierluigi Russo, Francesco Pio Bizzarri, Giovanni Battista Filomena, Filippo Marino, Roberto Iacovelli, Chiara Ciccarese, Luigi Boccuto, Mauro Ragonese, Filippo Gavi, Francesco Rossi, Cosimo Savoia, Paolo Pietro Suraci, Roberto Falabella, Savio Domenico Pandolfo, Luigi Napolitano, Chiara Leoni, Valentina Trevisan, Giuseppe Palermo, Marco Racioppi, Emilio Sacco, Stijn Muselaers, Nazario Foschi

**Affiliations:** 1 Department of Urology, Fondazione Policlinico Universitario Agostino Gemelli, IRCCS, 00168 Rome, Italy or pierluigi.russo@radboudumc.nl (P.R.); giuseppe.palermo@policlinicogemelli.it (G.P.); nazario.foschi@policlinicogemelli.it (N.F.); 2 Department of Urology, Università Cattolica Del Sacro Cuore, 00168 Rome, Italy; 3 Department of Urology, Ospedale Isola Tiberina—Gemelli Isola, 00168 Rome, Italy; 4 Department of Urology, Addenbrooke’s Hospital, Cambridge University Hospitals NHS Foundation Trust, Cambridge CB2 0QQ, UK; 5 Department of Urology, “F. Miulli” General Hospital, 70021 Acquaviva Delle Fonti, BA, Italy; 6 Department of Oncology, Fondazione Policlinico Universitario Agostino Gemelli, IRCCS, 00168 Rome, Italy; 7 Healthcare Genetics and Genomics, School of Nursing, Clemson University, Clemson, SC 29634, USA; 8 Section of Hygiene, University Department of Life Sciences and Public Health, Università Cattolica del Sacro Cuore, 00168 Rome, Italy; 9 Unit of Urology, Department of Medical-Surgical Sciences and Biotechnologies, Istituto Chirurgico Ortopedico Traumatologico Hospital, University of Rome Sapienza, Via F. Faggiana 1668, 04100 Latina, Italy; 10 Azienda Ospedaliera San Carlo, 85100 Potenza, Italy; 11 Department of Neurosciences, Reproductive Sciences and Odontostomatology, University of Naples “Federico II”, 80138 Naples, Italy; 12 Center for Rare Diseases and Birth Defects, Department of Woman and Child Health and Public Health, Fondazione Policlinico Universitario A. Gemelli, IRCCS, 00168 Rome, Italy; 13 Department of Urology, Radboud University Medical Center, 6525 GA Nijmegen, The Netherlands

**Keywords:** kidney cancer, testicular cancer, prostate cancer, bladder cancer, immune checkpoint inhibitors, Y chromosome

## Abstract

**Background:** The Y chromosome (ChrY) is essential for male sex determination and spermatogenesis. However, recent studies have revealed its broader role in various physiological processes and disease susceptibility, including cancer. **Methods:** A comprehensive literature review was conducted using databases like MEDLINE, Scopus, Web of Science, and Google Scholar. The review included clinical and preclinical studies in animals and humans focusing on the role of LoY in urological tumors. Data on the frequency of LoY, its clinical implications, and underlying mechanisms were extracted and analyzed. **Results:** The evidence suggests that LoY is associated with an increased risk of urologic neoplasms, potentially serving as an early marker of genomic instability. Studies reveal that LoY in urologic cancers correlates with worse survival outcomes and may contribute to tumor progression. LoY may interfere with chromatin structure and epigenetic regulation, suggesting its role as a contributor to early tumorigenesis. **Conclusions**: LoY appears to be a structural aberration with unique biological and clinical relevance in urologic cancers, possibly serving as a biomarker for genomic instability. Further research is necessary to identify specific Y-linked genes affected by LoY, potentially informing targeted therapies and early diagnostic strategies for these cancers.

## Simple Summary

This research examines how the loss of the Y chromosome (LoY) in men may contribute to the development and progression of certain cancers, particularly in the urinary system. Traditionally, the Y chromosome was primarily associated with male sex determination and sperm production, but recent studies suggest it plays a wider role in health and disease. LoY appears to disrupt immune function and increase genetic instability, factors that may heighten cancer risk and impact treatment outcomes. Our review highlights that LoY could serve as an early indicator of certain cancers and might help predict cancer behavior. Understanding LoY’s role could lead to new diagnostic tools and treatments, especially for cancers where Y chromosome alterations are linked to poorer outcomes.

## 1. Introduction

Aneuploidies are the presence of an abnormal number of chromosomes in a cell, which represent a hallmark of different cancer types. It is a common event that occurs at many stages of tumorigenesis [1]. The Y chromosome (ChrY) is unique to males and plays a role in determining sex, which stands out among all the human chromosomes due to its relatively small size and distinctively repetitive structure. This atypical configuration shields it from genomic degradation over time but also contributes to its alterations in gene copy number expression [2]. The ChrY, which is found only in males and determines sex, experiences mosaic loss (LoY) when it has lost cell subsets. This condition is a common occurrence in the somatic cells of elderly men. Because the ChrY physiologically does not have a “backup” copy, LoY leads to the loss of many unique and ubiquitously expressed genes, with potentially notable implications for cell fitness. Nevertheless, LoY shows a notably higher occurrence in tumor tissues and is linked to a poorer overall prognosis [3]. The root causes and subsequent effects of LoY are largely unidentified.

Among cancer patients, it is well established that men face an increased risk and worse prognosis compared to women [4]. Recent years have seen a heightened awareness of the biological disparities between sexes in cancer and the critical role of sex hormones in cancer development. This awareness has led to the identification of several loci on sex chromosomes associated with cancer vulnerability. In addition to sex hormone levels, both genetic and epigenetic factors in both males and females contribute to the clinical course of cancer [5]. While the influence of X chromosome-specific effects on cancer susceptibility in females has been extensively studied, the ChrY has historically been overlooked [6]. This negligence can be attributed to its limited gene content, the phenomenon of LoY in aging male cells, and its absence in females [2].

LoY represents the most prevalent acquired genetic alteration and a frequently encountered manifestation of clonal mosaicism in humans. A significant down-regulation of ChrY gene expression is linked to an increased risk of neoplasia in males. The functional outcome of LoY is represented in its gene expression, as suggested by proposed driver models [4].

It is now recognized that modifiable carcinogenic environmental exposures, such as arsenic, can induce LoY. The emerging use of LoY in blood as a biomarker offers significant promise in understanding the potential carcinogenic effects of environmental exposures [7]. Despite historical negligence in oncogenomics due to the additional steps required for copy number profiling, LoY is becoming more prevalent in tumor tissues within the cancer context [8,9,10].

Another relevant significant clinical implication is LoY association with diminished survival [11,12]. Patients with LoY in their blood were reported to experience an average reduction of 5.5 years in survival, making it a more accurate predictor of cancer prognosis than chronological age [13]. Several studies showed significant differences in the mutation frequency of oncogenes and tumor suppressor genes in LoY tumors compared to those with retained ChrYs. For instance, *TP53* mutations occur more frequently in LoY across certain cancers but less in others. The loss of *TP53* function correlates with the enrichment of somatic genome copy number alterations and poor prognosis in several cancer types [14], again indicating an association of LoY with the features of genomic instability [15]. Recent studies have found that *MMP13* (*Matrix Metallopeptidase 13*) and *MUC16* (*Mucin 16*) are significantly more frequently mutated in LoY across various cancer types. *MMP13* is involved in invasion, metastasis, and angiogenesis [16], while *MUC16* acts as an oncogene promoting proliferation and migration [17]. The enrichment of these mutations in LoY tumors suggests a more aggressive cancer biology.

In previous studies, several factors have been associated with mosaic LoY, such as age, exposure to environmental stressors, and genetic predisposition [7]. Initial cytogenetic analyses revealed that in male lymphocytes, the prevalence of mosaic LoY remains minimal up to the age of 15 years (0.05%), but it steadily rises to 1.34% in men between 76 and 80 years of age [18]. The large-scale population-based genome-wide association indicated that mosaic LoY in blood samples is less than 2% for men under 60, increasing to 15–40% of males aged 70–85 and affecting 57% of males by the age of 93 years [12,19,20]. In aging men, LoY can underlie negative consequences for human health; for example, recent epidemiological studies have observed an association with various forms of cancer [1], autoimmune diseases [21,22], senile maculopathy [23], cardiovascular disorder [24], Alzheimer’s disease [13], non-insulin-dependent diabetes [20], obese conditions, and general mortality [25]. It might be inferred then that age-related LoY in normal hematopoietic cells, resulting in aneuploidy, might be a hallmark of many cancer types [26,27]. For example, in bladder cancer, LoY was found in 30–40% of the cases [28,29] (Table 1). As recently reported by Abdel-Hafiz, Hany A et al. [30], in the bladder tumor microenvironment, the ability of cells with Y—is properly that of evading the host immune system through the cell exhaustion of CD8+ cells and thus the ability to grow much faster and be more aggressive, but at the same time an increased response to anti-PD1 ICI therapy.

Given the recognized importance of LoY in the pathogenesis, development, and mechanisms of tumor replication, we conducted a narrative review of the literature to provide an updated and comprehensive overview of the implications of LoY in various types of urologic cancers, encompassing the molecular mechanisms involved and extending to the clinical outcomes in terms of tumor incidence, prognosis, and overall survival, and their potential synergistic effect with another agent, such as ICI [39].

## 2. Resource and Approach

A bibliographic search was performed from inception to 31 August 2023 in several search engines/databases:

- MEDLINE (US National Library of Medicine, Bethesda, MD, USA);
- Scopus (Elsevier, Amsterdam, The Netherlands);
- Web of Science (Thomson Reuters, Toronto, ON, Canada);
- Google Scholar databases.

Different combinations of the following keywords were used according to a free-text protocol: “Y chromosome”, “loss of Y chromosome”, “urologic cancer”, “prostate cancer”, “kidney cancer”, “testicular cancer”, “bladder cancer”, “immunotherapy”, and “immune checkpoint inhibitors”.

Eligible articles were included, such as the following: only English language written on clinical (experimental or observational) or pre-clinical studies on humans or animals concerning the loss of the Y chromosome and its relationship with urological tumors. Conference abstracts, case reports, case series, and commentaries were excluded.

The research was performed by four independent reviewers (P.R., G.B.F., F.P.B., and N.F.), in two steps: title and abstract screening and full-text screening in order to assess the eligibility of the articles. After the screening phase, all the included articles were analyzed and the following data were extracted: bibliographic details (authors, year of publication, title, and journal); study design and population studied; methodologies used to detect LoY and its frequency in various types of urological tumors, the clinical and prognostic implications of LoY; and main results and conclusions.

The results were reported narratively due to the high heterogeneity of the included studies. A qualitative analysis was conducted to identify common themes, differences, and gaps in the existing knowledge. A quantitative synthesis (meta-analysis) was not performed due to methodological variability and the different outcomes reported in the studies.

## 3. Clinical Impact of LoY

### 3.1. LoY and Kidney Cancer

Renal cell tumors exhibit a heterogeneous morphology that can change during the process of carcinogenesis [36,40,41]. Molecular studies showed that LoY is detected in the non-malignant tubular epithelium in end-stage kidney disease (ESKD), a condition with an intrinsic higher likelihood of developing renal cell carcinoma [8]. Several types of renal cell tumors have been associated with ESKD, such as papillary renal cell carcinoma (pRCC, which occurs more frequently), clear renal cell carcinoma (ccRCC), and papillary adenoma [13,41,42]. Chromosome Y loss has also been reported in oncocytic neoplasms, particularly in chromophobe renal cell carcinoma and oncocytoma.

Büscheck et al. investigated the frequency and clinical relevance of LoY in kidney cancers by analyzing 1252 male renal tumors using tissue microarray format via fluorescence in situ hybridization (FISH) [43]. A LoY was detected in 47% of the analyzable male tumors, and its frequency correlated with the histologic subtype. The highest LoY frequency was most detected in pRCC (77%) compared to ccRCC (39%). Interestingly, the *MUC16* oncogene, which mediates proliferation and migration, and *MMP13*, implicated in invasion, metastasis, and angiogenesis, show higher mutation rates in LoY tumors of specific cancer types, such as renal papillary cell carcinoma [16,17].

In a study by Sadimin et al., a limited number of patients were examined, and no correlation was observed between high-grade mucinous tubular and spindle cell neoplasia (a rare and aggressive renal neoplasm) and LoY [44]. In pRCC, only lower tumor stages showed an increased LoY rate, with 88 patients exhibiting LoY tumors experiencing significantly fewer disease recurrences compared to 18 patients with tumors that retained ChrY. This suggests that LoY might be linked to a more favorable prognosis in papillary tumors.

Several genomic investigations on ccRCC have highlighted the importance of molecular irregularities that impede chromatin remodeling and epigenetic modifier function in ccRCC development [45]. Arseneault et al. investigated which chromosomal aberrations (germline and somatic) are more frequent in ccRCC. They found that tumors in males exhibited a greater frequency of recurrent chromosomal abnormalities, notably an increase in 7q (28% in males vs. 17% in females) and the deletion of 9p (25% in males vs. 10% in females) [36]. With respect to sex chromosomes, no tumors in male patients showed the loss of chromosome X. However, LoY was identified as the most prevalent somatic chromosome aneuploidy, occurring in approximately 37% of the cases and affecting the entire chromosome. Further confirmation of LoY’s role in ccRCC carcinogenesis comes from the down-regulation of the epigenetic modifiers *KDM5D* and *KDM6C* through somatic LoY [30].

A relationship exists between changes in ChrY copy number and the degree of aneuploidy, assessed using the total autosomal aneuploidy index (TAAI). It remains challenging to discern whether LoY is the causative factor or the consequence of high TAAI. Although LoY is closely tied to genome-wide instability, it might have unique implications. The presence of LoY in somatic non-cancer cells often correlates with large autosomal aneuploidy events, indicating that LoY may precede total autosomal aneuploidy [46]. These findings suggest that LoY might be an early-stage condition and could serve as a biomarker for predicting genome-wide instability [47].

Despite these findings, it is clear that LoY is evident in various histologic subtypes of renal neoplasms. The highest incidence occurs in pRCC, where it could serve as a significant prognostic biomarker and is related to better health outcomes. In metastatic ccRCC, Klatte et al. found that LoY is associated with improved progression-free survival in univariate analysis [48]. The cause of these differences remains unclear; however, evidence suggests that LoY’s impact on tumor biology may vary significantly across different cancer types. In chronic myelomonocytic leukemia, Y-loss is associated with a favorable outcome, whereas it is associated with a poorer prognosis in head and neck cancer and multiple myeloma, with no consequence observed in prostate and bladder cancer [49,50]. Finally, while there is a high prevalence of LoY in kidney cancer tumors, with varying rates among histologic subtypes, additional research is needed to determine the precise role of genetic modifiers in the development of renal neoplasia [43].

### 3.2. LoY and Testicular Cancer

Testicular cancer has increased during recent decades, mainly in industrialized countries, reaching its highest incidence among men aged 15 to 40 [51]. The risk factors associated are a family history of testicular germ cell tumors, testicular dysgenesis syndrome (cryptorchidism, hypospadias, reduced sperm production, and compromised fertility) or disorders/differences in gender formation [52,53,54], and 25 genome-wide association studies of susceptibility loci [55,56].

The ChrY, which is vital for testicular development and for initiating and sustaining spermatogenesis, contains many amplicons and palindromic sequences in its long arm (Yq) that might be subjected to deletions during spermatogenesis. In particular, the deletions of two genes, *DAZ* (*Deleted in Azoospermia*) and *AZF* (*Azoospermia Factor*), are strong candidates for male infertility [57]. In the *male-specific Y* (*MSY*) region, there are dense clusters of genes known as amplicon segments. These genes are present in multiple nearly identical copies and are predominantly or solely active in the testes, where they play a crucial role in supporting germ cell development and proliferation. Due to the significance of these genes, mutations within this region, coupled with the observation that individuals with testicular germ cell tumors (TGCTs) often have reduced fertility, suggest a potential link between deletions in the *AZF* region and the development of TGCT [10]. Moreover, the gr/gr deletion, a minor alteration on ChrY, has been associated with an increased risk of TGCT. It shows a stronger effect in familial TGCT cases (OR = 3.2) compared to sporadic instances (OR = 2.1) [9]. This deletion, recognized as an infrequent, low-penetrance allele, is linked to a heightened susceptibility to TGCT. Extensive European research has revealed that the gr/gr deletion independently raises the risk of TGCT, regardless of any changes in spermatogenesis. The loss of the *DAZ*, *BPY2*, and *CDY1* genes due to the gr/gr deletion suggests their potential suppressive roles in TGCT development [31,58].

Another study exploring the relationship between LoY and TGCT risk was conducted by Machiela MJ et al. They conducted two case–control studies, analyzing both blood- and buccal-derived DNA samples, to investigate whether mosaic loss across the entire ChrY correlated with TGCT [59]. Using quantitative polymerase chain reaction (qPCR), they assessed LoY by comparing the signal of the ChrY marker to that of the autosomal single-copy gene, RPPH1. Their findings revealed higher frequencies of mosaic LoY in familial TGCT cases compared to sporadic TGCT.

The ChrY has long been recognized as a potential factor in gonadal tumors. Individuals with testicular dysgenesis syndrome, even with small quantities of ChrY fragments, have an elevated risk of developing gonadal tumors, particularly gonadoblastoma [60,61]. A review by Kido et al. identified the human TSPY gene as a Y-linked gene with specific expression in the testis, hypothesized to play essential roles in male reproductive cell maturation, mitosis, and meiosis [38].

The TSPY protein has been detected in gonadoblastoma as well as different varieties of germ cell malignancies (GCTs). These include unclassified carcinoma in situ/intratubular germ cell neoplasia, which is the precursor for all TGCTs, seminomas, and extragonadal intracranial GCTs [62,63]. Beyond GCTs, TSPY is often observed in somatic tumors, such as liver neoplasm [64], melanoma [65], and prostate neoplasia [66]. TSPY expression is more prevalent in CIS and gonadoblastoma, particularly during the initial stages of germ cell tumorigenesis, suggesting a pivotal role in carcinogenesis, particularly in the early germ cell lineage. Consequently, TSPY-positive cases are more prone to GCT development or progression than TSPY-negative cases. Understanding the role of TSPY expression in cancer progression, its potential as a biomarker, and its relevance for targeted therapy requires further analysis, including clinical outcomes alongside TSPY [67].

While the ChrY plays a crucial role in regulating spermatogenesis, there is limited data regarding the prevalence of testicular neoplasms in men with Yq microdeletions. Urgent attention is warranted due to the unstable nature of the deleted ChrY, which results in a significant loss of genetic material. This loss may predispose individuals to testicular GCTs.

### 3.3. LoY and Prostate Cancer

Prostate cancer (PCa) stands as the second most frequently diagnosed cancer in men, with a prevalence of 59% in the eighth decade [68]. The incidence of the condition varies significantly across geographical regions, with higher rates observed in countries with robust screening programs [69,70]. The risk factors encompass genetic predisposition (including positive family history or genetic conditions like Lynch syndrome or *BRCA1* and *BRCA2* pathogenic variants), metabolic syndrome, and various dietary, exogenous, or environmental factors [71]. The investigations of chromosomal aberration in PCa could play a role in the analysis of origin and progression. In fact, considering genetic factors, more than 100 loci may predispose to the risk of developing PCa. The most common germline mutations affect the *BRCA2* (13q12.3), *ATM* (11q22.3), *CHEK2* (22q12.1), *BRCA1* (17q21), *HOXB13* (17q21.2), and *MMR* genes [72]. In recent years, genomic research has focused on the ChrY, investigating its association with PCa and revealing a correlation between LoY and PCa development. Despite the link between LoY and PCa, some studies indicate that the LoY is confined to the prostate epithelial tissue where the tumor is situated, with no corresponding loss observed in the surrounding tissue, including epithelium or stroma tissue [73].

**Figure 1:**
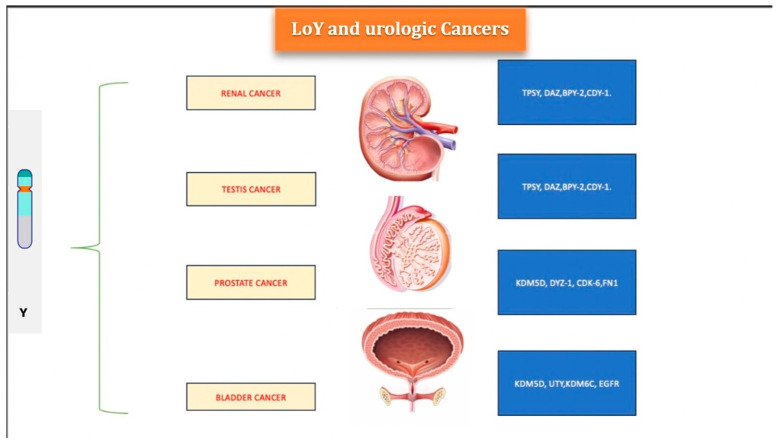
Image illustrating the main urological tumors associated with the loss of the Y chromosome and their respective genes.

König et al. demonstrated that LoY is a frequent chromosomal aberration in PCa that is most often found as the only abnormality with a frequency of 53%. However, despite the elevated percentage, it is difficult to think that there is a correlation exclusively with PCa, as LoY also reflects the states of non-pathological tissue hyperproliferation [74]. (Figure 1) A study conducted by Jordan et al. [75] showed a correlation between PCa and LoY. They demonstrated that not only does LoY not always correlate with PCa, but also that the regional loss of LoY is associated with the origin of the tumor in PCa tissue and adjacent epithelium histologically showing benign prostatic hyperplasia (BPH). Although BPH is not considered a malignant disease in this study, a correlation between LoY and the future progression of PCa is considered highly plausible. The detection of LoY in prostatic intraepithelial neoplasia (PIN) and BPH supports this theory [76,77]. Noveski et al., on the other hand, investigated LoY’s role in PCa development in peripheral blood cells, confirming its association with PCa but without confirming a correlation with aging. This contrasts with the established mechanism of LoY tied to telomere shortening and genomic instability as age advances [20].

*AZF* genes, expressed in many organs outside the testis, have drawn particular interest. Several studies have reported the male-specific *histone lysine demethylase 5D* (*KDM5D*) gene as a tumor suppressor in PCa. *KDM5D* plays a role in regulating transcriptional mechanisms and the cell cycle. Its loss is associated with invasive metastasis formation, more aggressive PCa, and a poorer prognosis. Additionally, it influences PCa sensitivity to docetaxel through its interaction with androgen receptor (AR) signaling. Nadal reported LoY in the seminal vesicles of 28 PCa patients [35,78,79,80]. Khosravi et al. identified 51 sequence-tagged sites on the ChrY related to the male-specific region. They also noted variations in both the sequence and copy number of the *DYZ1* gene in LNCaP cells (which are androgen-dependent and metastasize to lymph nodes) and DU145 cells (which are androgen-independent and originate from brain metastasis) PCa. Bashir et al. found that Malaysian men diagnosed with PCa had four Y-linked short tandem repeats (STRs): *DYS388*, *DYS435*, *DYS437*, and *DYS439*, all situated on the *DYS* locus [33].

They conducted a study comparing the Y-chromosomal haplogroups of 92 Japanese PCa patients with those of 109 randomly selected healthy Japanese male controls. The males were categorized into four haplogroups: DE, O2b*, O2b1, and an untagged group, based on three binary Y-chromosome markers—the sex-determining region Y (SRY), YAP, and 47z. Their findings revealed that males with DE and the untagged haplogroups exhibited a higher risk of developing PCa. Further evidence of the malignant impact of LoY comes from murine experiments. These studies demonstrated that introducing the ChrY into a LoY PCa cell led to reduced tumor formation in a xenograft model [81].

In recent years, several long noncoding RNAs (lncRNAs), including *SChLAP1*, *HOTAIR*, *PCA3*, *PCAT1*, *NEAT1*, and *CTBP1-AS*, have emerged as key regulators in various physiological and pathological processes, particularly in the initiation and progression of PCa [82]. A study by Xiao G. et al. revealed that the highly up-regulated Y-chromosomal lncRNA *TTTY15* plays a pivotal role in promoting PCa. This effect is attributed to *TTTY15* acting as a molecular sponge for the microRNA *let-7*, resulting in the increased expression of *CDK6* and *FN1* [32]. Notably, *FN1* holds significance in cancer metastasis, while *CDK6* governs cell proliferation [83,84]. A significant up-regulation of *TTTY15* was evident in the majority of PCa samples. Given its monoallelic nature on the ChrY, *TTTY15* holds a distinct advantage for gene editing therapies. Thus, *TTTY15* emerges as a promising candidate with potential therapeutic implications for a subset of PCa patients.

Nowadays, contemporary efforts to identify genetic alterations associated with LoY and others have led to the development of several computational tools, such as the GECIP toolbox. These tools are instrumental in analyzing these alterations and their effects on pathways, particularly in prostate cancer research. Understanding mutations, copy number alterations, and gene expression changes is pivotal in cancer research, as it aids in identifying underlying causes, heterogeneity within tumors, and mechanisms of drug resistance [85].

### 3.4. LoY and Bladder Cancer

Urothelial bladder cancer causes significant cancer-associated mortality globally, with a higher incidence in Europe and North America, and it ranks as the 7th most frequently diagnosed cancer in males but falls to the 10th position when considering both genders [86,87,88]. The risk factors include smoke, occupational exposure, environmental pollution, sex, race, and genetic predisposition. Recent research, besides the well-known genetic conditions like Lynch syndrome or Costello syndrome [89,90,91], has pinpointed specific genetic loci associated with bladder cancer risk, with this cancer boasting the third-highest mutation frequency after melanoma and lung cancer [92]. In addition to bladder cancer-related genes like *TP53*, *RB1*, *FGFR*, *PIK3CA*, *HRAS*, *KRAS*, and *TSC1* [93], changes in genes controlling chromatin remodeling and chromatid cohesion and segregation have also been identified [94]. As tumors develop and progress due to the accumulation of genomic changes, studying these alterations could offer deeper insights into bladder tumor biology and potentially yield improved prognostic indicators.

Understanding the impact of cytogenetic changes on tumor development and progression remains a challenge in bladder cancer research. LoY, observed in 10–40% of the cases [28,29], represents one such enigma. While LoY is clearly linked to bladder cancer, the precise mechanisms driving this connection remain incompletely understood [95,96].

One possible explanation for the connection between LoY and bladder cancer lies in exposure to harmful factors, notably smoking. Studies by Dumanski et al. have shown that current smokers have a higher prevalence of LoY compared to non-smokers. Interestingly, the effect of smoking on LoY appears to be dose-dependent. However, unlike bone marrow cells, where LoY increases with age, LoY in bladder cancer seems unrelated to patient age. Notably, normal urothelium in males of all ages retains the ChrY, suggesting a distinct pattern of genetic alterations in bladder cancer [97].

Sandberg et al. reported that men with bladder cancer who exhibit LoY tend to have a poorer prognosis compared to those whose tumors retain the ChrY [98]. Additionally, most tumors with LoY show concomitant cytogenetic changes, often indicative of more advanced disease stages [99].

While some studies have not found a direct link between LoY and tumor features like grade, stage, or specific gene (such as *p53*) mutations [96], others suggest LoY tumors might be more aggressive [29,50,100]. Recent research by Hafiz Abdel et al. provides some insight [30]. They showed how LoY and related genes like KDM5D and UTY can weaken the immune system’s response to tumors, making them more aggressive. Their findings (first in mouse models and later in human bladder tumor samples) suggest that LoY tumors create an environment that suppresses the immune system, especially affecting CD8+ T cells. Interestingly, these T cells from LoY tumors respond better to anti-PD1 treatment, hinting at potential new therapies [101]. In clinical practice, detecting the loss of *KDM5D* and *UTY* could serve as valuable prognostic indicators, aiding in assessing the clinical aggressiveness of bladder cancer and guiding treatment decisions. Understanding the complex relationship between LoY and tumor behavior is key to developing targeted therapies and improving patient outcomes.

Another study by Kobatake et al. using a murine model suggests a different therapeutic approach [37]. They found that missing genes on both X and Y chromosomes (*KDM6A* and *KDM6C*) contribute to bladder cancer. The absence of these genes triggers the pathways involved in inflammation (IL-6 and CCL2), which in turn fuels cancer growth. Interestingly, this process works together with mutations in the *TP53* gene, further accelerating tumor development. Based on this, the researchers propose using the existing drugs to target these inflammation pathways. Drugs like the anti-human IL6 receptor antibody Tocilizumab (for rheumatoid arthritis) and the CCR2 inhibitor Propagermanium (for hepatitis B) could be repurposed for bladder cancer treatment. This approach holds promise for a new type of targeted therapy, potentially for other cancers driven by inflammation as well.

A study by Caceres et al. showed that the significant down-regulation of ChrY gene expression acts as a male-specific marker for cancer susceptibility. This is closely associated with LoY, ChrY methylation patterns, and environmental factors like smoking. The study suggests that certain genes, such as *DDX3Y*, *EIF1AY*, *KDM5D*, *RPS4Y1*, *UTY*, and *ZFY*, play a role in protecting women from many types of cancer compared to men [33,60]. Between them, we can find bladder cancer that is strongly associated with this condition. These genes are located on a specific region of the Y chromosome (*NRY*) and act as tumor suppressors in a dose-dependent way, influencing cell division. Men are more prone to mutations in these genes than women due to a biological process called X-inactivation. Additionally, factors like *Epidermal Growth Factor Receptor* (*EGFR*) gene activity can lead to the increased methylation (silencing) of *NRY* genes in certain cancers [34].

While LoY itself might not affect the tumor growth stage, Abdel-Hafiz’s work suggests that LoY tumors can evade the immune system [30]. Their findings demonstrated that these variants possessed the ability to escape adaptive immunity by inducing CD8+ T cell fatigue. This immunological phenomenon contributes to immune dysfunction within the neoplastic context, further enhancing the tumor’s response to ICI. This underscores the potential significance of assessing the expression of ChrY genes, particularly *UTY* and *KDM5D*, as a means of identifying bladder cancer patients who may benefit from ICI therapy in order to achieve improved response rates and enhanced survival outcomes [102].

Furthermore, there is a speculative potential for temporarily inhibiting *UTY* and *KDM5D* pharmacologically, which could provide enhanced therapeutic benefits for patients with ChrY-high tumors undergoing PD-1/PD-L1 immune checkpoint blockade. However, further research is needed to clarify the underlying mechanisms responsible for LoY-driven tumor evasion. Additionally, the precise identification of the molecular pathways linking the loss of *UTY* and/or *KDM5D* to CD8+ T cell fatigue within the tumor microenvironment is crucial for advancing our understanding and developing targeted therapeutic strategies.

### 3.5. LoY and Immune Response

The role of ChY is also becoming increasingly important in several oncological fields due to its likely relationship with the immune system and complex polygenic traits. As mentioned earlier, LoY has been associated with several diseases (hypertension, coronary artery disease, autoimmunity diseases, and cancer) that share an altered immune system response. A possible explanation is the theory of “the immunosurveillance hypothesis” in which the LoY in blood cells disrupts the normal immune function of leucocytes, leading to the onset of disease in the various organs where immune cells are distributed [103].

Hollows et al. showed the difference in immune gene activity in the male head and neck squamous cell neoplasia in cases with or without LoY, and the results were that the LoY cells had overexpression of genes with functions in the oxidation process, including those associated with resistance to both radiotherapy and cisplatin-based chemotherapy and a down-regulation of genes with the functions of immune response and inflammatory response [3,104]. Males negative for HPV with LoY exhibit notably shorter overall survival compared to those without LoY. In addition to an elevated Tumor Mutation Burden (TMB), specific genomic alterations can influence tumor behavior, prognosis, and the response to ICI.

Similar findings emerged in bladder cancer. Recent research suggests that LoY is linked to a poorer prognosis and the expression of immune T cells [30]. These “exhausted” T cells are less effective at fighting cancer. Interestingly, when researchers removed the ChrY in mice with bladder tumors, they saw even more dysfunctional T cells. These tumors also responded better to an immune checkpoint blocker, and this association was consistently observed in datasets [30,104], and this gains importance in bladder cancer therapy.

Considering all this evidence, we can suppose that ChrY plays a role in the immune response and has tumor suppression functions. This theory is further supported by research showing that reintroducing a ChrY can suppress tumors in mice with prostate cancer [82]. Considering this, functional studies have verified the genes that could be crucial for male viability: *RPS4Y*, *ZFY*, *TBL1Y*, *PRKY*, *USP9Y*, *DDX3Y*, *UTY*, *TMSB4Y*, *NLGN4Y*, *CYorf15A*, *KDM5D*, and *EIF1AY* [105]. In particular, two of those, *UTY* and *ZFY*, seem to have a tumor suppression function, as evidenced by Davoli et al. [106], while they examined the distribution of tumor suppressor genes across sex chromosomes. Both genes have counterparts on the X chromosome that evade X chromosome inactivation. *UTY* has the *UTX* homolog (also known as *KDM6A*) that encodes a demethylase of lysine 27 on histone 3 (H3K27), which is linked to dysregulated squamous cell differentiation in HNSCC cell lines: *UTY* could have the same function albeit with reduced activity [107,108]. This explains how the ChrY performs tumor suppressor activity along with X-linked and other autosomal genes.

Cancer development can be considered as the escape of cancer cells from the surveillance of the immune system, resulting in the failure of immune response (a process defined as immunoediting) [109]. Conversely, genomic instability is associated with acquiring a malignant phenotype and the subsequent metastatic spread [110]. These two major events, deeply interconnected, are the backbone of carcinogenesis in advanced-stage tumors.

## 4. Conclusions

In this review, we emphasized the association between LoY and the increased risk of developing urologic neoplasms. LoY may arise within the framework of general genomic instability, where it is likely a passenger, lost due to its small size and limited gene content [11]. Genomic research on urologic cancers has stressed the significance of molecular abnormalities that hinder the operation of chromatin restructuring and epigenetic modulators in cancer development [2,37]. Several findings suggest that LoY may represent an early-stage indicator of disease and could act as a biomarker for forecasting genome-wide instability. Previous research have indicated a shorter overall survival for patients with LoY-associated tumors. Some other studies propose that LoY increases the likelihood of cancer development in males and contributes to cancer progression. This aligns with the association of LoY with adverse outcomes and implies that LoY could be a preliminary event in tumorigenesis. Future studies will shape the knowledge about which Y-linked genes are down-regulated and this can lead to an earlier diagnosis of genetic conditions associated with these mutations. This insight might pave the way for the development of targeted therapies to tackle the dysregulated molecular pathway in these conditions and define the likelihood of transmitting such altered genes to an offspring. In conclusion, our review reveals that LoY constitutes a structural anomaly in individuals affected by urologic cancers [45]. This aberration holds unique biological and clinical importance, emphasizing the need for in-depth explorations and additional research endeavors.

## Figures and Tables

**Table 1:** Informative table of genes implicated in various urological tumors and their respective cellular functions and cancer-related effects.

Study	Gene/Gene Family	Functional Domain	Function	Expression	Correlation with Cancer
**Moreno-Mendoza et al., 2019 [31]**	BPY-2	Y-linked gene (AZFc region)	cell cycle regulation	Testis (human tissue)	GCTs
**Xiao G et al., [32]**	CDK-6	Y-chromosomal lncRNA TTTY15	cell cycle regulation	Prostate (human tissue)	PCa progression and metastasis
**Moreno-Mendoza et al., [31]**	CDY-1	Y-linked gene		Testis (human tissue)	GCTs
	DAZ	Y-linked gene (AZFc region)	cell cycle regulation	Testis (human tissue)	GCTs
**Khosravi et al., [33]**	DYZ-1	Y-linked gene	cell cycle regulation	Prostate (human tissue)	Lymph node involvement
**Xiao G et al., [32]**	FN1	Y-chromosomal lncRNA TTTY15	cell cycle regulation	Prostate (human tissue)	PCa progression and metastasis
**Dunford et al., [34]**	EGFR	Y chromosome non-recombining region (NRY)	cell cycle regulation	Bladder (human)	Bca susceptibility
**Marino et al., [35]**	KDM5D	Y-linked gene (AZFc region)	transcriptional mechanisms and cell cycle	Prostate (human tissue)	Poor prognosis in PCa, metastasis formation.
**Abdel-Hafiz et al., [30]**	KDM5D	Y-linked gene	regulation of immunity (CD8 cell regulation)	Bladder (murine and human tissue)	BCa aggressiveness
**Arseneault et al., [36]**	KDM5D	Y-linked gene	cell cycle regulation	Renal cell (human tissue)	Carcinogenesis in kidney cancer (ccRCC)
	KDM6C	Y-linked gene	cell cycle regulation	Renal cell (human tissue)	Carcinogenesis in kidney cancer (ccRCC)
**Kobakate et al., [37]**	KDM6C	Y-linked gene	regulation of cytokine and chemokine pathways (IL-22 and CCL2)	Bladder (murine)	Bca development
**Kido et al., [38]**	TSPY	Y-linked gene	cell cycle regulation	Testis (human tissue)	Gonadoblastoma and various types of GCTs.
**Abdel-Hafiz et al., [30]**	UTY	Y-linked gene	regulation of immunity (CD8 cell regulation)	Bladder (murine and human tissue)	BCa aggressiveness

## Data Availability

The original contributions presented in the study are included in the article, further inquiries can be directed to the corresponding author/s.

## Abbreviations

y chromosome (ChrY)
loss of y chromosome (LoY)
germ cell tumors (GCTs)
testicular germ cell tumors (TGCTs)
prostate cancer (PCa)
male-specific histone lysine demethylase 5D (KDM5D)
androgen receptor (AR)
short tandem repeats (STRs)
sex-determining region Y (SRY)
long noncoding RNAs (lncRNAs)
Y chromosome non-recombining region (NRY)
Epidermal Growth Factor Receptor (EGFR)
using co-detection indexing (CODEX)
total autosomal aneuploidy index (TAAI)
